# Inadequate Access to Potable Water Impacts Early Childhood Development in Low-Income Areas in Cape Town, South Africa

**DOI:** 10.5334/aogh.4281

**Published:** 2023-11-24

**Authors:** Caradee Y. Wright, Thandi Kapwata, Caylee Cook, Steven J. Howard, Hleliwe Makaula, Rebecca Merkley, Mbulelo Mshudulu, Nosibusiso Tshetu, Natasha Naidoo, Gaia Scerif, Catherine E. Draper

**Affiliations:** 1 Environment and Health Research Unit, South African Medical Research Council, Pretoria, ZA; 2 Department of Geography, Geoinformatics and Meteorology, University of Pretoria, Pretoria, ZA; 3 Environment and Health Research Unit, South African Medical Research Council, Johannesburg, ZA; 4 Faculty of Health Sciences, Department of Environmental Health, University of Johannesburg, Johannesburg, ZA; 5 SAMRC-Wits Developmental Pathways for Health Research Unit, Department of Pediatrics, Faculty of Health Sciences, University of the Witwatersrand, Johannesburg, ZA; 6 Early Start and School of Education, University of Wollongong, Wollongong, NSW, AU; 7 Department of Cognitive Science, Carleton University, Ottawa, CA; 8 Department of Experimental Psychology, University of Oxford, Oxford, UK

**Keywords:** climate change, child health, education, environmental health, IDELA, mental health, vulnerability

## Abstract

**Background:**

Water and sanitation are vital to human health and well-being. While these factors have been studied in relation to health, very little has been done to consider such environmental risk factors with child development. Here, we investigated possible relations between household water access/storage and early childhood development in four low-income settlements in the City of Cape Town, Western Cape province of South Africa. Our objectives were 1) to determine water access/storage practices in dwellings of children; 2) to assess early childhood development; and 3) and to understand the relationship between water access/storage practices in relation to early childhood development.

**Methods:**

We used a questionnaire to assess household water risk factors and the International Development and Early Learning Assessment (IDELA) tool to assess child early learning / cognitive, socio-emotional and motor development.

**Results:**

Mean age of the children (N = 192) was 4 years and 55% were female. The mean IDELA score was 48% (range: 36–54%) where the higher the score, the better the child’s development. Around 70% of households had a tap inside their dwelling and half said that they stored water with the largest percentage of storage containers (21%) being plastic/no lid. Child IDELA scores were lower for children living in households that did not have an indoor tap and for households who stored water.

**Conclusions:**

Given the risks associated with climate change and the already poor conditions many children face regarding water and sanitation, research is needed to further investigate these relations to provide evidence to support appropriate interventions and ensure healthy child development.

## 1. Introduction

According to global estimates, in 2017 unsafe water sources caused 1.2 million deaths, accounting for 2% of global deaths and 6% of deaths in low- to middle-income countries (LMICs) [1]. Microbiologically contaminated drinking water can transmit diseases such as cholera, dysentery, typhoid, and polio and is estimated to cause almost 500,000 diarrhoeal deaths globally each year [2]. Insufficient availability of safe drinking water has a greater impact on the most vulnerable members of society, such as children, pregnant women, the elderly and people with pre-existing diseases. Water shortages disproportionately affect children [3]. In 2020, 26% of the global population did not have access to a safely managed service located on their premises, available when needed and free from contamination [2]. Inadequate access to potable water exposes children to various waterborne diseases, malnutrition, and dehydration [4]. In low-income settlements, households rely on communal taps or standpipes for water access, which are often unreliable and contaminated [5]. Therefore, providing safe and clean water to vulnerable populations, especially children, is crucial for ensuring their health and well-being.

There are few studies that explore the effect of inadequate water consumption or lack of access to potable water on child cognitive, socio-emotional, and motor development. There is some research on the relationship between water intake and cognitive development, but the overall body of literature in this area is relatively small. A German study found that increased water consumption during school hours had a positive impact on short-term memory and cognitive ability of children [6]. A study on pre-adolescents found that habitual water intake had a positive impact on cognitive flexibility, with a 34% deficit in working memory of children in the low water intake group [7]. These studies were carried out in high-income settings and show that even with easy access to water, there needs to be improved awareness of the importance of regular hydration in children.

Potable water and safe sanitation are important in LMICs as they affect childhood development and health. A large multicentre study involving India, Peru, Ethiopia, and Vietnam found that insufficient water and sanitation significantly affects childhood language development, thus having additional adverse impacts beside infectious disorders and stunting [8]. A meta-analysis of studies in LMICs showed a correlation between lack of water and sanitation and poor cognitive and motor development in children [9]. In Brazil, increased access to piped water and sanitation facilities led to higher levels of educational attainment as measured by years of schooling [10]. Among 20 sub-Saharan African countries, there was a positive correlation between improved drinking water (OR = 1.07, 95% CI 1.00 to 1.14, p = 0.046) and “on-track” cognitive development [11]. Given the critical role of safe water in child health and development, further research is necessary to explore the effects of inadequate access to potable water on children’s developmental outcomes. Furthermore, children residing in households with limited access to safe water sources are at an increased risk of experiencing anaemia, stunted growth, and wasting, which also lead to cognitive impairments [12].

Important public health and policy implications would result from an association between children’s development in water-scarce regions and the availability of drinking water [13]. This study seeks to redress the inattention to how environmental risks related to water are potentially affecting early childhood cognitive, socio-emotional, and motor development in South Africa. To that end, we investigated possible relations between household water access and storage and early childhood development in low-income settlements in and around the City of Cape Town, Western Cape province. No previous studies were retrieved that looked at water quality and child cognitive and social development or health impacts in this province. One study investigated the association between pesticide exposure (not water) and neurodevelopment in rural farmlands in the Western Cape province [14].

The aim of the study was to assess early child cognitive, socio-emotional, and motor development in relation to household water access and storage. Specific objectives were 1) to determine water access and storage practices in dwellings of children; 2) to assess early childhood development using a tool (discussed below) that covers multiple cognitive, motor, and social development domains; and 3) and to understand the relationship between water access and storage practices in relation to early childhood development (considering our broad understanding of its multiple domains). The data collected in this study will provide baseline knowledge on household water access and storage and early childhood learning and development among children living in the Western Cape province.

## 2. Methods

### Study design

This study was part of a larger short-term longitudinal study in which data collection occurred over two time points, that is, time point 1 and time point 2. The data from the current study were from time point 2 (i.e., the second round of data collection) when a selection of measures (not the full set) was included.

### Study sites

Between 2020 (time point 1) and 2021 (time point 2), participants were recruited from four low-income communities (Sites A, B, C and D) within the Cape Town Metropolitan area. In low-income settlements in and around Cape Town, South Africa, many households struggle with water access and storage [5]. Two of the communities (Site A and B) were classified as an urban township and were made up of mostly informal housing. Overcrowding is an issue in these communities with population densities of about 16,000 per km^2^ and 10,000 per km^2^. Other challenges include high rates of unemployment, food insecurity, alcohol abuse, crime and human immunodeficiency virus / acquired immune deficiency syndrome. The other two communities (Site C and D) were part of what is referred to as the Cape Flats and were made up of both formal and informal housing. The population density varies within the suburbs from which participants were recruited (between 4,000 per km^2^ and 16,000 per km^2^). Gang activity and drug abuse are major challenges in these community in addition to high rates of unemployment, crime, and food insecurity [15].

### Participants

This study included 192 children aged 3–5 years and their primary caregiver from low-income settings in Cape Town. Specifically, children who were not attending early childhood care and education (ECCE) services were recruited as they represent a particularly vulnerable group that remains understudied, and constitute a significant proportion (30%) of the population of 3–5-year-old children [16]. Children not attending any form of ECCE in South Africa are likely at a higher risk for poor developmental outcomes, potentially have higher levels of exposure and are consequently influenced more by the experiences within the home and community environment due to increased time spent in these environments (and not in ECCE services) [17].

Recruitment of children and their primary adult caregiver (aged >18 years) was facilitated through our partner organisation who implement a home-based stimulation programme for pre-school age children and their families living in environments characterised by poverty, unemployment, crime, and violence. Due to COVID-19 restrictions in place at the time, initial recruitment took place telephonically. Contact numbers of interested caregivers were provided by the organisation’s home visitors and passed on to the study’s research assistants who provided the potential participants with information about the study, answered any questions, invited caregivers to participate, and obtained informed consent.

The initial recruited sample at time point 1 was 243 caregiver/child dyads. Due to the hard lockdown during the COVID-19 pandemic, some of the first caregiver interviews were conducted telephonically. Of those, 11% of the child data were lost as we were not able to contact the caregiver to conduct the child testing in person at time point 1. At time point 2, 22% of the caregiver/child dyads were lost to follow-up. The reasons for loss to follow-up included difficulties contacting the caregivers (due to changing phone number or losing their phone) or caregivers moving out of the community and therefore no longer with the community organisation. Participants lost to follow-up did not differ significantly in child age, caregiver age, caregiver education, household assets, exposure to violence, or the home learning environment compared with those participants who participated in the full study. Demographic data that were missing at the time point 2 were inferred from demographic data available at the first time point. Additional missing data were very low (<2%) and therefore no further data imputation was done.

### Measures: Parent/caregiver questionnaire

The parent/caregiver questionnaire collected demographic details including the child’s age and birthweight, languages spoken in the home, number of adults and children living in the home, caregiver education, and child education. It also collected data about child exposure to stressful life events, caregiver adjustment and practices, home learning environment, household income, impacts of COVID-19, and water security and safety. The caregiver questionnaire was designed to be administered in approximately 30 minutes.

### Measures: Water-related household questions

The World Health Organization gathers information on water access and storage in households and reports these at country level [18]. The same variables and similar responses (tailored for South African conditions) were used for the water-related questions in this questionnaire. The questions included: main source of drinking water (tap inside dwelling, outdoor tap on site, communal tap offsite, water tanker/truck, other); storage of water for drinking or cooking in a container (no; yes), if yes, information on the type of container, size of opening, lid or no lid, means of making and keeping stored water clean (boil, bleach, other), frequency water storage container is washed, how water is removed from storage container (utensil such as scoop, cup, jug, other) and if there is one utensil that everyone uses to remove water from the storage container. We also asked, “in the last 4 weeks, how frequently did you or anyone in your household worry you would not have enough water for all your household needs?” This was asked to gauge reliability of access to water.

#### Measures: International Development and Early Learning Assessment (IDELA)

The IDELA tool is a rigorous, open-access, global tool to assess children’s early learning and development between the ages of 3.5 to 6 years (i.e., preschool age group) [19]. The IDELA tool was developed by Save the Children in 2011, following a comprehensive review of existing child development assessments and was informed by existing tools that were adapted to include a balance of internal applicability in low-resource settings, feasibility, ease of use, and scientific rigor [19,20].

The materials needed for the assessment are low-cost (i.e., pencils, blank papers, counting items, printable picture cards and a child’s book) and can be adapted for each country according to the available and contextually appropriate resources. The IDELA tool is comprised 24 main items that assess four key developmental domains namely: emergent math / numeracy (number sense, shapes, sorting, problem solving, comparison, simple operations), emergent language and literacy (print awareness, expressive vocabulary, letters, phonological awareness, listening comprehension), social-emotional development (empathy, emotional awareness, self-awareness, solving conflict, peer relationships), and motor skill development (hopping, copying a shape, folding paper, drawing). Answers for each item were marked as either correct, not correct, or no response. The scores for each of the four subdomains and for the total score vary from 0% (when the child did not answer the items correctly) to 100% (when the child answered the item correctly).

For the current study, IDELA was translated into isiXhosa and Afrikaans and used along with the English version. Children were tested individually by trained research assistants with their caregiver present. Testing was in the child’s home in the preferred language and took approximately 30 minutes per child. Consultations with the research team and the IDELA community of practice revealed that the original version of the tool was contextually appropriate and no further adaptations beyond the translation of the task were needed.

### Procedures

All testing was conducted by three research assistants who were trained on the procedures and the assessments and could speak the home language of the participants (i.e., IsiXhosa, English or Afrikaans). Due to COVID-19 restrictions and that some of the areas that participants were recruited from posed a safety risk to the research assistants, testing took place in various ways. For two of the areas, child testing and caregiver questionnaires took place in the dwelling of the participants at a time that was suitable for the participant. For the other two areas that posed a safety risk for the research assistants, a central venue was organised, and participants (caregiver and child) were given transport money to come to the testing venue. All tests were explained to caregivers and children so that they knew what was expected of them. Child participants completed the IDELA task as well as additional assessments that were included as part of the larger study. If there was more than one child in the household that qualified to participate in the study, a separate questionnaire was administered for each child. Caregivers received grocery vouchers for completing the questionnaire and again for bringing their child to testing. Children received stickers for their participation.

All data were captured on Redcap and transferred into Stata 15.1 [21], where quality control and data cleaning were performed prior to data analysis. The procedures for this study were approved in advance by the Human Research Ethics Committee (Medical) of the University of the Witwatersrand (Reference: M200104).

### Data management and statistical analyses

Data analysis was conducted using Stata 15.1 [21], and a p-value < 0.05 was considered statistically significant for all tests. Univariate regression models were used to determine unadjusted associations between source of water and IDELA scores, and water storage and IDELA scores of children from the four study sites. Multivariable regression was conducted to estimate adjusted associations between source of water and IDELA scores, and water storage and IDELA scores. Models were adjusted for covariates including age and sex of the child, number of people living in the home, and household income range.

## 3. Results

### Sample descriptives

Among the 192 child participants, the mean age was four years and the majority were female (55%) (Table 1). Most households had a monthly income below ZAR 6,000 (USD 310 in June 2023). Households comprised on average six members. The mean IDELA score by area ranged between 36% to 54% with an overall mean of 48%—the higher the score, the better the child’s development. We did not present the finding by sub-scores due to the relatively small sample size.

**Table 1:** Descriptive of sample (N = 192) by age, gender, mean household number of individuals, mean household income and IDELA scores by study area and all areas.


	SITE A n = 39 n (%)	SITE B n = 49 n (%)	SITE C n = 72 n (%)	SITE D n = 32 n (%)	TOTAL n = 192 n (%)

*Age*					

3 years	0 (0)	2 (4)	0 (0)	0 (0)	2 (1)

4 years	14 (36)	15 (31)	15 (21)	10 (31)	54 (28)

5 years	20 (51)	27 (55)	37 (51)	22 (69)	106 (55)

6 years	5 (13)	5 (10)	20 (28)	0 (0)	30 (16)

*Gender*					

Male	19 (49)	24 (49)	31 (43)	13 (41)	87 (45)

Female	20 (51)	25 (51)	41 (57)	19 (59)	105 (55)

*Household income*					

R750 or less	2 (6)	1 (3)	7 (10)	3 (10)	13 (8)

R750–R1,500	11 (31)	5 (14)	25 (36)	7 (24)	48 (28)

R1,500–R3,300	13 (36)	11 (31)	29 (41)	10 (33)	63 (37)

R3,300–R6,000	9 (25)	13 (38)	8 (12)	9 (30)	39 (23)

R6,000–R11,000	1 (2)	4 (11)	1 (1)	1 (3)	7 (4)

R11,000–R27,000	0 (0)	0 (0)	0 (0)	0 (0)	0 (0)

R27,000 or more	0 (0)	1 (3)	0 (0)	0 (0)	1 (1)

Missing	3 (–)	14 (–)	2 (–)	2 (–)	21 (–)

Mean individuals per household	5	5	6	6	6

Mean IDELA score (%) (n = 189, 3 missing)	50	54	49	36	48


### Water-related household findings

Around 70% of households had a tap inside their dwelling providing them with water (Table 2). Around half said that they stored water, and the largest percentage of storage containers (21%) were plastic and totally open (with no lid). Most people who stored water did not treat the stored water and those who did either boiled it or added bleach. Around 54% of participants reported that they washed the water storage container either daily, or once or twice a week. Participants storing water mainly used a utensil to decant water from the storage container. Two-thirds of participants reported worrying (mostly rarely or sometimes) about not having enough water for all household needs in the past month.

**Table 2:** Findings from the water-related questions in the questionnaire by area and for all areas (N = 192).


	SITE A n = 39 n (%)	SITE B n = 49 n (%)	SITE C n = 72 n (%)	SITE D n = 32 n (%)	TOTAL n = 192^#^ n (%)

*Where do you mainly get your drinking water from?*					

Tap inside dwelling	14	35	62	22	133 (71)

Outdoor tap on site	8	1	4	7	20 (11)

Communal tap-offsite	16	9	3	1	29 (16)

Private water seller	0	0	0	0	0 (0)

Water tanker/truck	1	0	0	0	1 (1)

Borehole	0	0	0	0	0 (0)

Rainwater tank	0	0	0	0	0 (0)

River/stream/dam	0	0	0	0	0 (0)

Other	0	0	0	0	5 (x)

Missing		0	3	2	4 (–)

*Do you store your water for drinking or cooking in a container? (e.g., bucket or 5 litre container)*					

Yes	7	29	37	17	90 (54)

No	32	13	16	15	76 (46)

Missing					26 (–)

*If you store water, is the container:*					

Metal, totally open	0	0	0	0	0 (0)

Metal, small hole-too small to get fist in	0	0	1	0	1 (0.5)

Metal, large hole-big enough to get hand in	0	0	0	0	0 (0)

Plastic, totally open	8	5	8	11	32 (21)

Plastic, too small to get fist in	7	1	5	4	17 (11)

Plastic, large hole-big enough to get hand in	7	5	2	1	15 (10)

Other, totally open	10	0	0	0	10 (7)

Other, small hole-too small to get fist in	1	1	0	0	2 (1)

Other, large hole-big enough to get hand in	1	0	0	0	1 (0.5)

Not applicable	2	20	37	16	75 (49)

Missing					39 (–)

*If you store water, does the container have a lid?*					

Yes	3	4	2	6	15 (17)

No	31	12	16	13	72 (83)

*What do you do to the water to make it and keep it clean?*					

Nothing	33	34	39	18	134 (72)

Boil the water	3	12	22	11	48 (26)

Add bleach	3	1	0	0	4 (2)

Add disinfectant fluid	0	0	0	0	0 (0)

Add chlorine	0	0	0	0	0 (0)

Other	0	0	0	0	0 (0)

*If you store water, how often do you wash your water storage container?*					

Never	4	17	24	9	54 (41)

Monthly	2	1	1	0	4 (3)

Once or twice a week	14	3	8	4	29 (22)

Daily	15	8	7	12	42 (32)

Other	0	1	0	1	2 (2)

*If you store water, how do you take water out of the container?*					

Using a utensil (scoop, cup, jug)	34	18	17	18	87 (84)

Hands	0	0	0	0	0

Other	1	3	6	6	16 (16)

*If a utensil is used, is there one special cup that everyone uses for this?*					

Yes	18	8	4	4	34 (68)

No	17	15	19	22	73 (32)

*In the last 4 weeks, how frequently did you or anyone in your household worry you would not have enough water for all your household needs?*					

Never	13	27	57	21	118 (62)

Rarely (1–2 times)	14	11	9	7	41 (21)

Sometimes (3–10 times)	11	9	6	0	26 (14)

Often (11–20 times)	1	1	0	3	5 (3)

Always (more than 20 times)	0	0	0	1	1 (<1)

Missing	0	1	0	0	1 (–)


*Note*: ^#^ Questions pertaining to storage of water do not use N = 192 as the denominator because the total number of people answering the question varied for each question.

### Regression results

Unadjusted univariate regression results showed that the predicted IDELA scores for children from Site B, where participants reportedly got their water from outdoor taps on the stand, were 34 points lower than those who had access to taps inside the dwelling (Table 3). This association was found to be statistically significant (p = 0.042). However, the conference interval was wide. The relationships between the main source of water and IDELA scores for children from Site A, Site C and Site D were not statistically significant.

**Table 3:** Unadjusted multiple regression results for relationship between IDELA score and main source of water for children living the four study sites.


STUDY SITE	CHARACTERISTIC	CATEGORY	CO-EFFICIENT	P-VALUE	95% CONFIDENCE INTERVAL

Site A	Main Water Source	Tap inside dwelling*	–	-	–

		Outdoor tap on site	10.51	0.154	–4.13–25.15

		Communal tap-off site	–2.12	0.723	–14.22–9.97

	Storage of water	Yes	1.81	0.797	–12.40–16.02

		No*	–	–	–

Site B	Main Water Source	Tap inside dwelling*	–	–	–

		Outdoor tap on site^$^	–34.05	0.042^#^	–66.80–1.29

		Communal tap-off site	–1.17	0.847	–13.30–10.97

	Storage of water	Yes	–8.31	0.125	–19.03–2.41

		No*	–	–	–

Site C	Main Water Source	Tap inside dwelling*	–	–	–

		Outdoor tap on site	–9.25	0.440	–33.04–14.54

		Communal tap-off site	–18.17	0.132	–41.96–5.61

	Storage of water	Yes	1.01	0.872	–11.51–13.53

		No*	–	–	–

Site D	Main Water Source	Tap inside dwelling*			

		Outdoor tap on site	4.73	0.454	–8.05–17.52

		Communal tap-off site	9.40	0.527	–20.73–39.54

	Storage of water	Yes	10.05	0.036^#^	0.67–19.43

		No*	–	–	–


*Note*: ^#^ refers to statistically significant category because p < 0.05.

Univariate analysis also found that storage of water was statistically significant in Site D, where the IDELA scores were calculated as being higher for children who lived in homes that reported storing water for domestic purposes (co-efficient = 10.06, p = 0.036) compared to those who did not. Once again, the confidence interval was wide likely due to the relatively small sample size.

After adjusting for potential confounding factors, that is, age and sex of the child, number of people living in the home, and household income range for each of the sites, IDELA scores among children from Site B were lower for those from households who stored water compared with those that did not store water (co-efficient = –12.13, p = 0.042, 95% CI = [–23.74] – [–0.52]) (Table 4).

**Table 4:** Adjusted multiple regression results for relationship between IDELA score and main source of water for children living in the four study areas.


STUDY SITE	CHARACTERISTIC	CATEGORY	CO-EFFICIENT	P-VALUE	95% CONFIDENCE INTERVAL

Site A	Main Water Source	Tap inside dwelling*	–	–	–

		Outdoor tap on site	9.83	0.273	[–8.31]–27.97

		Communal tap-off site	2.33	0.746	[–12.40]–17.05

	Storage of water	Yes	–1.15	0.911	[–22.15]–19.86

		No*	–	–	–

Site B	Main Water Source	Tap inside dwelling*	–	–	–

		Outdoor tap on site^$^	–	–	–

		Communal tap-off site	8.47		

	Storage of water	Yes	–12.13	0.042^#^	[–23.74]–0.52

		No*			

Site C	Main Water Source	Tap inside dwelling*	–	–	–

		Outdoor tap on site	–17.59	0.171	[–43.07]–7.89

		Communal tap-off site	–21.44085	0.079	[–45.45]–2.57

	Storage of water	Yes	5.72	0.436	8.97–20.40

		No*	–	–	–

Site D	Main Water Source	Tap inside dwelling*			

		Outdoor tap on site	–10.32731	0.308	[–31.05]–10.40

		Communal tap-off site	–18.77088	0.329	[–58.19]–20.65

	Storage of water	Yes	18.12597	0.084	[–2.73]–38.98

		No*			


*Note*: Models adjusted for age and sex of the child, number of people living in the home and household income range, * refers to reference category, ^$^ refers to an omitted category, ^#^ refers to statistically significant category because p < 0.05.

## 4. Discussion

This study showed that while most participants had a tap inside their dwelling for water access, many households relied on an outdoor tap on their stand/plot of land. This despite the government’s goal of providing all dwellings with adequate water and sanitation services, including an indoor tap. Majority of the participants had a relatively low monthly income only slightly above the monthly upper-bound poverty line of ZAR 1 415 per person per month [22] bearing in mind that households comprised 3 to 8 people.

### IDELA scores

The IDELA scores for the children in our study were lower compared to those of Brazilian children (participant ages were 3 and 6 years) living in Sao Paulo [23]. Our mean score was 48% (range 36–54%) while it was 78% (74–89%) for the Brazilian study participants. Possible reasons for these differences are socio-economic status and schooling conditions—children in the Brazilian study may have been attending school while our child participants were not attending school.

### Relations between water access/supply and IDELA scores

Properly treated water provided to indoor taps is likely to be cleaner than water from an outdoor tap (potentially shared between several people) that must be collected and brought indoors [24,25]. The IDELA scores for children from Site B were most affected by source of water, that is, indoor tap or outdoor tap. This was the one site with a statistically significant association where children from households where water was obtained from outdoor taps on the stand had lower IDELA scores compared with children who had access to an indoor tap. Access to an indoor tap is easier than having to walk outside, perhaps in inclement weather, to fetch water for consumption. Moreover, an indoor tap is likely more “visible” and a reminder to drink water when one is thirsty compared to when a tap is outdoors. It is also likely that having an indoor tap versus having an outdoor tap is correlated with socio-economic status and income level; this site is known to have a range in household income levels.

### Water storage and child development

Water storage is common in South African households due to the risk of non-continuous water supply or the need to treat the water because the supplied water is deemed unclean [26]. Several households stored water, likely because of water service interruptions, to ensure continuous access to water. In Site D, 73% of homes used water from a tap in the home and 53% stored some of that water. When making use of an indoor tap, water may be obtained and consumed immediately or stored for later use. When using an outdoor tap, water must be collected in a container and transferred into the dwelling. The container may be unclean, and the water may become contaminated between the point of collection and the place of indoor storage. Moreover, people and animals may drink water from the outdoor tap transferring contaminants from mouth to tap. An indoor tap is less likely accessible to animals and water is more likely to be poured into a container/cup for consumption keeping the tap (relatively) clean compared to a tap from which people drink water directly.

Site B had a statistically significant association in the adjusted (for age and sex) regression where IDELA scores were calculated to be lower for children from homes where water was stored. Only half the participants reported washing their water containers – an essential practice for safe water storage. Several reflections arise in relation to these water storage and child development findings. Water storage may have been perceived differently by households, for example, storing drinking water in a refrigerator versus storing water in a 10-litre drum without a lid. In this site, several households made use of outdoor taps on their stand or a communal outdoor tap. Future studies should be specific about water storage possibilities. It is also possible that water storage is an indicator of socio-economic status and this status varied widely in Site B.

The City of Cape Town regularly imposes water restrictions to keep the water system running until heavy rains occur [27]. This suggests that our study sites are vulnerable to the impacts of climate change; water storage is an important part of climate change adaptation therefore communities need to understand how to safely store water. For example, most homes that stored water did not cover the container, and stored water was not treated prior to drinking. The World Health Organization’s Household Water Treatment and Safe Storage Manual [28] advises that to keep water safe, best practices include treating water (by bringing water to a rolling boil) prior to storage, cleaning the storage container regularly, covering the container with a lid, and not contaminating (for example by drinking from) the scoop or utensil used to decant water from the storage container. Such knowledge should be shared with all households who store water to ensure safe, adequate water in places with water restrictions or non-continuous water supply for the benefit of child development, as well as reduction of health outcomes, such as diarrhoeal disease [29]. It is important to note that access to electricity or alternative fuel sources for boiling water may be a challenge for people living in low-income communities and alternatives may be needed, for example, solar thermal water disinfection [30].

### Study strengths and limitations

Few studies have considered IDELA scores in relation to environmental factors and we consider our study among the first to do so in relation to water. A Brazilian study linked child development to community noise exposure at the child’s home and found that community noise exposure may impair behavioural and cognitive development of preschool children [23].

Another strength of this study was the parent/caregiver and child dyads that allowed us to gather multiple variables around water access and storage in households in relation to child development. Despite the relatively small sample due to loss to follow-up, we consider our results to be robust. We did not test water quality in any of the participants’ homes and this should be considered for future studies. It would also be helpful to ask about household sanitation since water and sanitation are closely linked. Future studies may also want to consider additional development tests, such as the Early Learning Measures Outcome (ELOM) [31] to support interpretation of the IDELA scores. We used a cross-sectional study design, and one might consider an alternate design such as a cohort or prospective study to gain further insight into the relationship between water access/storage and child development. Finally, we did not conduct an analysis on IDELA sub-scores to consider differential effects on these different domains but future studies with larger samples should consider doing so.

## 5. Conclusions

Our study found that having access to water via a tap inside the dwelling was associated with good child development of 3–5-year-old children compared to only having access to water via a tap outside the dwelling on the stand/plot. There were conflicting findings related to the storage of water: in some household where water was stored, child development was poor while in others where water was stored, child development was good. In the instance where water storage practices were good, for example, containers were likely sealed, washed regularly and the water was treated, it is likely that the water quality was fair while being stored. Stored water also implies that children have access to drinking water, even when the water services delivering water via taps is non-operational, temporarily or for an extended period. It would be beneficial to raise awareness among communities about best practices for water storage, as described above, to support general health but also ensure that children have access to clean water with the benefit of potentially improved child development. Parents and caregivers also require more knowledge about the benefits of encouraging children to drink water regularly. Local authorities should support improvement of adequate, safe water service provision in both formal and informal settlements. Periodic water quality testing would also benefit the community to ensure proper operation of water treatment works and provision of safe, potable water. Additional studies on the relationship between water and child development are needed to bolster the findings from this study in South Africa and elsewhere.

## Data Accessibility Statements

All authors had access to the data and a role in writing the manuscript.
